# Data-driven determination of number of discrete conformations in single-particle cryo-EM

**DOI:** 10.1016/j.cmpb.2022.106892

**Published:** 2022-05-16

**Authors:** Ye Zhou, Amit Moscovich, Alberto Bartesaghi

**Affiliations:** a Department of Computer Science, Duke University, Durham, NC 27708, USA; b Department of Statistics and Operations Research, Tel Aviv University, Tel Aviv, Israel; c Department of Biochemistry, Duke University School of Medicine, Durham, NC 27708, USA; d Department of Electrical and Computer Engineering, Duke University, Durham, NC 27708, USA

## Abstract

**Background and objective:**

One of the strengths of single-particle cryo-EM compared to other structural determination techniques is its ability to image heterogeneous samples containing multiple molecular species, different oligomeric states or distinct conformations. This is achieved using routines for in-silico 3D classification that are now well established in the field and have successfully been used to characterize the structural heterogeneity of important biomolecules. These techniques, however, rely on expert-user knowledge and trial-and-error experimentation to determine the correct number of conformations, making it a labor intensive, subjective, and difficult to reproduce procedure.

**Methods:**

We propose an approach to address the problem of automatically determining the number of discrete conformations present in heterogeneous single-particle cryo-EM datasets. We do this by systematically evaluating all possible partitions of the data and selecting the result that maximizes the average variance of similarities measured between particle images and the corresponding 3D reconstructions.

**Results:**

Using this strategy, we successfully analyzed datasets of heterogeneous protein complexes, including: 1) in-silico mixtures obtained by combining closely related antibody-bound HIV-1 Env trimers and other important membrane channels, and 2) naturally occurring mixtures from diverse and dynamic protein complexes representing varying degrees of structural heterogeneity and conformational plasticity.

**Conclusions:**

The availability of unsupervised strategies for 3D classification combined with existing approaches for fully automatic pre-processing and 3D refinement, represents an important step towards converting single-particle cryo-EM into a high-throughput technique.

## 1. Introduction

One of the strengths of single-particle cryo-EM compared to other experimental techniques for structure determination is its ability to successfully deal with sample heterogeneity and the inherent conformational flexibility of large protein complexes [1-4]. While techniques such as principal component analysis (PCA), covariance matrix estimation [5-8], diffusion maps [9, 10], and algorithms based on deep neural networks have recently been proposed to solve the continuous heterogeneity problem [11-15]-, approaches that produce a discrete number of classes (K) are still widely used in the field and have proven to be very effective at characterizing structural variability in cryo-EM datasets [3, 4]. This strategy for classification is implemented in most software packages for cryo-EM data analysis [16-19], and consists of partitioning particle projections into a discrete number of homogenous subsets, each representing a different conformation. The number of clusters K used to partition the dataset, however, is a parameter specified by the user that may not always reflect the underlying distribution of the data and could potentially introduce bias and lead to inaccurate conclusions [11, 20, 21]. Moreover, a single round of 3D classification is rarely sufficient to characterize the complete landscape of conformations present in a dataset, and multiple classification rounds are usually needed to find the correct partition, thus increasing the computational requirements of this ad-hoc procedure.

With the advent of automated strategies for high-throughput data collection and the availability of faster cameras [22, 23], a single TEM instrument can nowadays generate tens of thousands of exposures per day [22,24-28]. To keep up with this rate of data production, streamlined and efficient strategies to analyze large collections of images are needed that can routinely convert raw data into 3D structures. Indeed, several software packages for cryo-EM data analysis already implement automated routines for on-the-fly processing that are useful for monitoring data quality and allow users to quickly assess sample quality and improve the efficiency of data collection and instrument utilization [29-31]. Many of these packages have successfully automated the initial steps in the cryo-EM image processing pipeline, including movie frame alignment, CTF estimation, particle picking and 3D reconstruction. While progress has also been made in the automated selection of good quality micrographs [32], automated identification of “good” 2D class averages [32], and unsupervised particle sorting during 3D refinement [33], strategies to solve the 3D heterogeneity problem still remain largely supervised, thus creating a bottleneck in the cryo-EM structure determination pipeline. While general strategies for estimating the number of classes in K-means clustering, such as the gap statistic [34], have been proposed, these methods are based on the Euclidean distance between data points (or particle images in our case), which in cryo-EM are not available because observations correspond to 2D projections taken from random orientations, preventing the application of these methods.

Here, we propose an unsupervised approach to automatically detect the number of discrete conformations present in single-particle cryo-EM datasets. Our strategy consists in executing a series of clustering steps using increasing values of K followed by an analysis of key statistics resulting from each 3D classification run. In particular, we show that maximizing the average variance of similarities measured between particle projections and each 3D class allows us to automatically find the value of K that best captures the number of discrete conformations present in the data, without the need for user intervention. To validate our approach, we first analyzed datasets created by artificially combining micrographs from different datasets of antibody-bound HIV-1 Env trimers. We also analyzed several datasets downloaded from the EMPIAR database containing different levels of intrinsic heterogeneity. In all cases, we were able to show that our strategy successfully recovered the correct number of clusters. Our approach represents another step towards streamlining the structure determination pipeline in single-particle cryo-EM, paving the way to convert this technique into a high-throughput strategy for studying heterogeneous protein complexes at high-resolution.

## 2. Methods

One of the main challenges in achieving accurate 3D classification in single-particle cryo-EM is that image alignment parameters are unknown and are dependent on the 3D references determined during refinement. Existing strategies for analyzing continuous flexibility all assume that a single consensus alignment solution is available, potentially biasing the classification results [13,35]-[37]. To overcome this problem, we adopt the simple, yet effective solution of systematically carrying out multiple classification runs using increasing values of K, followed by statistical analysis of the results. Similar to existing algorithms for 3D classification of cryo-EM images, we first execute an ab-initio alignment step where all extracted particles are aligned to an external reference using projection matching. This provides an initial set of consensus alignment parameters that we use as initialization to run multiple simultaneously rounds of 3D classification, each time increasing K by one. While this brute-force strategy may seem computationally prohibitive, the fact that orientation searches are done locally, significantly speeds up the refinement process while ensuring that particle alignments are optimized for each partition of the data. Unlike existing algorithms that assume a single set of particle alignments prior to running the variability analysis, our approach guarantees that each image will be correctly aligned to its corresponding reference, thus resulting in more accurate 3D classification results and also higher resolution 3D reconstructions.

After executing multiple runs of classification on several datasets, we observed that once the value of K exceeded the actual number of conformations present in a mix, a low-resolution class containing low quality particles appeared (from now on termed “noisy” class). This phenomenon has been observed before, and the interpretation is that these classes act as sinks that attract particles that do not match any of the other classes [18]. To detect the presence of the noisy classes, we analyzed “score” values produced by the 3D refinement package cisTEM [16]. Score values represent the quality-of-fit between each experimental raw image and re-projections from the reconstructions produced during 3D classification, and consists of a scaled version of the cross-correlation plus a term to restrain the spatial alignment parameters. Following projection-matching refinement, score values against each reference are assigned to every raw particle in the dataset:

$$s_{k,i}=score(R_{k},I_{i}),$$

where $R_{k}$ is the k-*th* 3D reference and $I_{i}$ is the i raw particle image. After each 3D classification round, we parsed the score values $s_{k,i}$ produced by cisTEM and estimated the number of clusters present in a discrete mix by maximizing the average score variance:

$$K_{best}=argmax_{K}\frac{\sum_{k=1}^{K}\frac{1}{N_{k}}\sum_{i=1}^{N_{k}}\;(s_{k,i}-\underline{s_{k}}{)}^{2}}{K}$$

where $\underline{s_{k}}=\frac{1}{N_{k}}\sum_{i=1}^{N_{k}}s_{k,i}$ is the score-mean of particles assigned to class k, and $N_{k}$ is the number of particles assigned to class k. K typically varies between 1 and $K_{max}$, which in practice rarely exceeds $K_{max}=10$ due to the size limitations of single-particle datasets and the requirement of having a sufficient number of particle images in each class [13, 14].

**Fig. 1.**
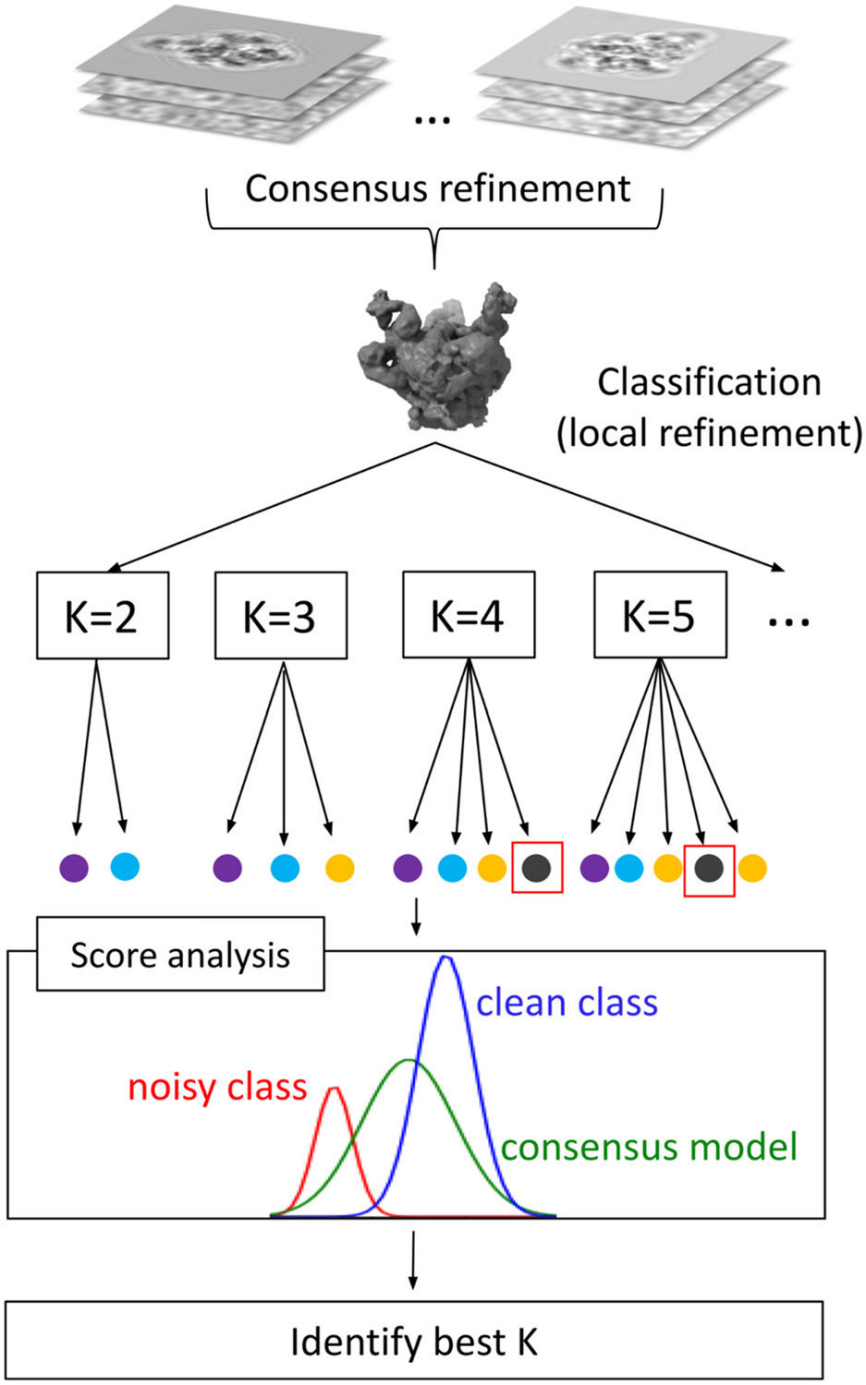
Data-driven strategy for determining the number of conformations in cryo-EM. Starting from a dataset containing images from a discrete number of conformations, we first derive a consensus model by refining particle alignment parameters against a single reference. We then carry out a series of 3D classification runs using increasing values of K and analyze the resulting cisTEM score statistics (“Score analysis” box). When K=1 (consensus model), scores show a unimodal distribution (green curve). For values K>1, we detect the presence of either noisy classes (low scores, red curve), or clean classes (high scores, blue curve). For values of K higher than the actual number of classes present in the dataset, we observe the appearance of noisy reconstructions (indicated as black filled circles surrounded by red boxes). To detect this change, we use the mean of cisTEM score variances as a robust criteria to automatically determine the number of distinct conformations present in the mix.

The expression for $K_{best}$ is based on the empirical observation that the partition with the broadest spread of scores is the one that best captures the variability in the dataset. In general, the appearance of “noisy” classes generates score distributions with lower mean and variance (since poor quality particle images have lower correlation with the noisy references), contributing to an overall reduction in the mean score variance. When the value of K is smaller than the number of conformations present, mixing of particles from different species will occur, leading to reduced resolution and narrower average score distributions. For K larger than the actual number of conformations, noisy classes will appear and uniform populations of particles would be subdivided into smaller classes, also leading to narrowing of the average variance of cisTEM scores. Based on these observations, our strategy to determine the number of conformations in a discrete mix consists in maximizing the mean score variance over a range of K values, Figure 1.

## 3. Results

We adopted a two-step approach for validating our strategy. First, we analyzed synthetic mixtures of single-particle images which provide ground-truth for accurately evaluating performance. We also analyzed intrinsically heterogeneous datasets downloaded from the Electron Microscope Public Image Archive (EMPIAR) [38], chosen to represent different types of heterogeneity and varying levels of difficulty in terms of class separability, Supplementary Table 1.

### 3.1. Unsupervised identification of antibody-bound gp120 HIV-1 Env trimer species

**Fig. 2.**
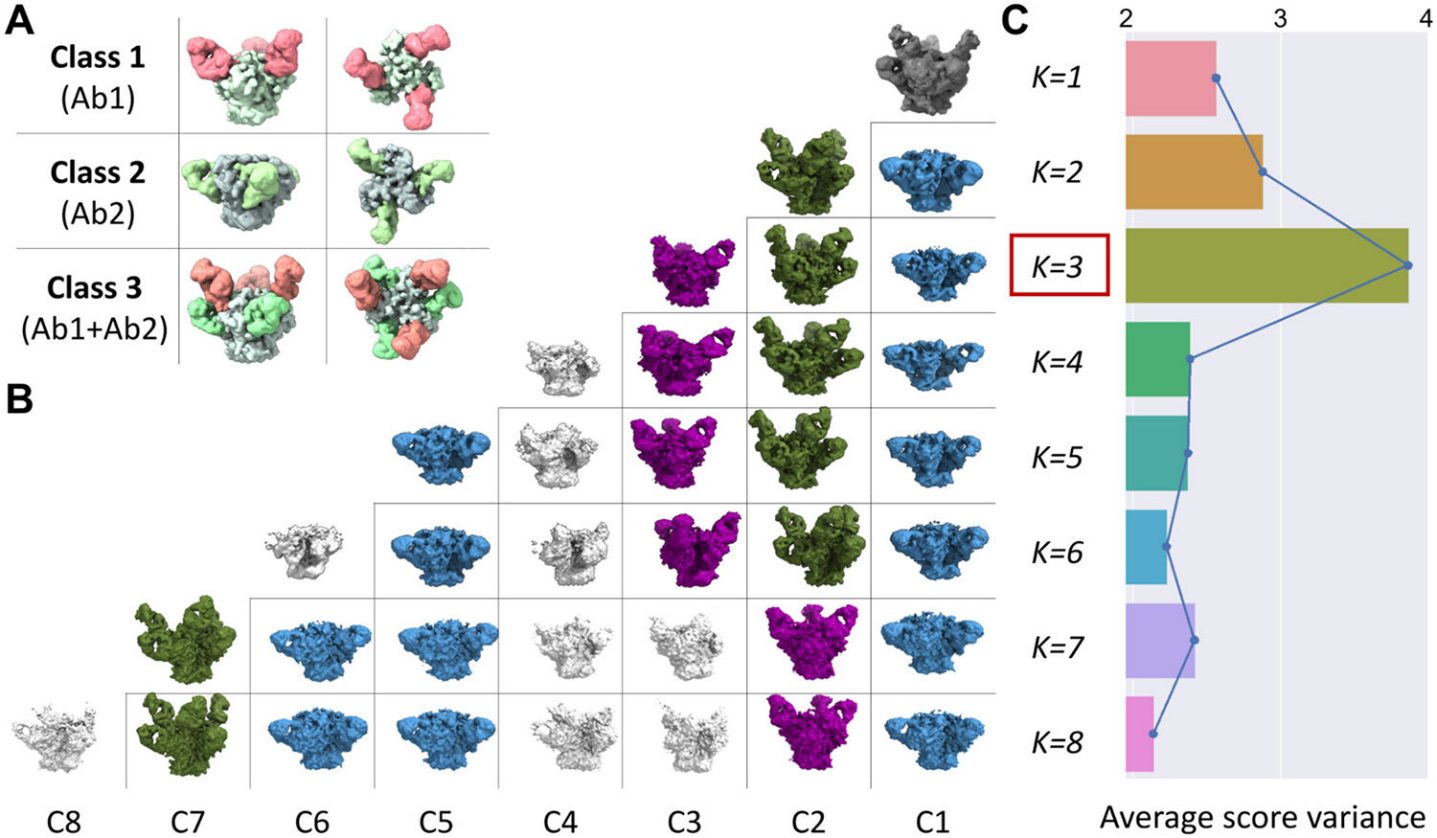
Automatic determination of the number of Ab-bound HIV-1 Env conformations. **A.** Upper left panel shows the three different original conformations corresponding to the HIV-1 Env (gp120-gp41 trimer) bound to Ab1 (Class 1), Ab2 (Class 2) and to Ab1 plus Ab2 simultaneously (Class 3). Density for Ab1 is shown in light red and density for Ab2 is shown in light green. **B.** Incremental classification results are shown as a triangular matrix with each row representing 3D classification results obtained for values of K ranging between 1 (consensus model) and 8. Reconstructions are color coded according to their structural identity: Class 1 (blue), Class 2 (purple), Class 3 (dark green) and Noisy classes (gray), which appear only for K>3. **C.** Distribution of average cisTEM score variances for each value of K. Red box highlights the peak occurring at K=3, coinciding with the correct number of HIV-1 Env species present in the heterogeneous mix. The unmasked estimated resolutions of the maps are 7.7 Å (Class 1), 6.7 Å (Class 2), and 6.9 Å (Class 3).

The trimeric HIV-1 Envelope protein (Env) mediates viral-host cell fusion with allosteric elements in each protomer orchestrating host receptor-induced exposure of the co-receptor binding site and fusion elements. Each Env monomer is a complex of the gp120 and gp41 glyco-proteins. Studying structural heterogeneity of antibody-Env complexes is a powerful tool to provide insights on Env conformation that are relevant for vaccine design [39-41]. Here, we simulated heterogeneous datasets by combining particles extracted from micrographs of homogeneous samples of HIV-1 gp120-gp41 trimers bound to different antibodies. While the overall conformation of the gp120-gp41 trimer was similar in all the datasets we analyzed, different antibodies with distinct epitopes and angles of approach were considered (Figure 2A and Supplementary Figure 1A). After combining 50,000 particles from each dataset into a single concatenated stack, we first executed a round of consensus refinement where all particles were iteratively aligned against a common 3D reference (without using classification). We then ran multiple 3D classification rounds using values of K ranging from 2 to 8, using only local orientation and translational searches for each particle to speed up calculations. Despite the high degree of similarity between the different antibody-bound complexes, our results show that the 3D classification procedure can successfully separate particles and reveal their original identities, both, when combining 2 datasets (Supplementary Figure 1B) or 3 datasets (Figure 2B). Interestingly, every time the value of K was increased beyond the true number of clusters present in the dataset, a lower quality “noisy” reconstruction was consistently observed, and further increases in K resulted in the appearance of additional noisy classes and subclasses corresponding to subsets of the original conformations. To analyze the statistics from the 8 classification runs, cisTEM score values reflecting the quality of the match between each 3D reference and the particle images were extracted for each value of K. The mean score variance was maximized when K matched the actual number of clusters (Figure 2C and Supplementary Figure 1C). This metric therefore serves as a reliable indicator to detect the number of discrete conformations present in a mix.

### 3.2. Automatic identification of conformations of the calcium-selective channel TRPV5

We also tested our routines on a second dataset that was generated by combining 100,540 particles of the calcium-selective channel TRPV5 with calmodulin bound (EMPIAR-10256) and 66,071 particles from the closely related TRPV5 mutant W583A (EMPIAR-10253) [42], Supplementary Figure 2A. The first dataset contains wild type TRPV5 and calmodulin (CaM) mixed in vitro, and the reported 3D reconstruction shows strong density of both the N-lobe and C-lobe of CaM binding at one corner of the TRPV5 channel. The mutant dataset contains TRPV5-W583A without manual addition of CaM, but still has the ability of binding endogenous CaM from expressing cells. The mutation disrupts CaM C-lobe binding while leaving the N-lobe unaffected. The main difference we found between the 2 classes is in the C-lobe density (shown in pink in Supplementary Figure 2A). These datasets are challenging because the extra density corresponding to calmodulin is very small and breaks the symmetry of the complex making accurate particle alignments critical to achieve a successful separation. After conducting 3D classification using values of K between 1 and 6 (Supplementary Figure 2B), we observed a similar behavior as in the HIV-1 Env cases where a lower quality class consistently showed up after the number of clusters increased beyond the true number of conformations. The mean score variance values derived from the cisTEM scores (Supplementary Figure 2C), were maximized at K=2, allowing us to automatically identify the correct number of conformations. We also observed that having an additional noisy class can actually improve the resolution of the reconstructions due to the removal of lower quality particles that get assigned to the noisy class. Indeed, one can leverage this in order to obtain better quality maps, by first estimating $K_{best}$ and then using the 3D maps from the $K_{best}+1$ run.

### 3.3. Unsupervised classification of the calcium homeostasis modulator (CALHM) chimera

Next, we tested our approach on a more challenging case of an intrinsically heterogeneous dataset that contains a discrete number of classes within the same set of images. For this experiment, we used particles extracted from the EMPIAR-10444 entry, corresponding to the calcium homeostasis modulator (CALHM) chimera. This dataset contains 2 discrete classes with 8 (8-mer) or 9 (9-mer) protomers as reported in the original study [43]. Using our strategy to determine the optimal value of K, we carried out 3D classification analysis using 1-5 classes and identified K=2 as the optimal value. The partition we obtained, correctly captured the 8-mer and 9-mer classes, as well as a noisy class appearing when K=3, Supplementary Figure 3. Despite the subtle differences between the protomers, the average score variance in this case also clearly peaked at K=2. As before, given the symmetry mismatch between the classes, accurate determination of orientations was critical to correctly classify this dataset.

### 3.4. Towards a fully automatic pipeline for single-particle cryo-EM data analysis

**Fig. 3.**
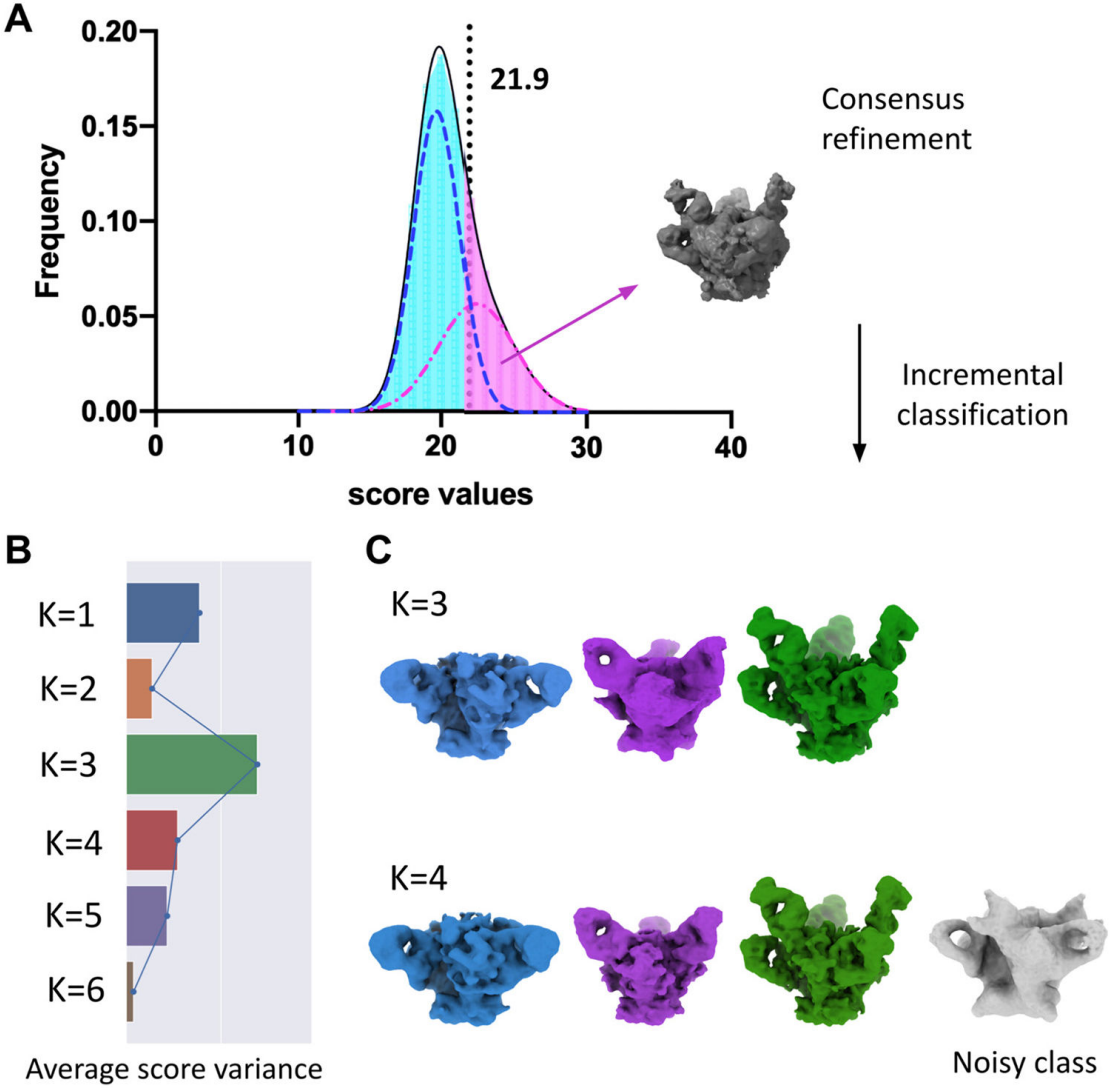
Fully unsupervised end-to-end workflow for 3D classification of HIV-1 Env datasets. **A.** Distribution of cisTEM scores corresponding to the raw set of particles extracted from micrographs representing a mix of three antibody-bound conformations, and calculated against a consensus 3D model (gray). A bimodal Gaussian mixture model was fitted to this distribution to automatically identify the set of clean particles (pink, semi-dashed curve) that were consistent with the consensus model [44], and the set of lower quality particles (blue, dashed curve). Only particles belonging to the pink area (those to the right of the intersection between the modes) were kept and used for subsequent classification rounds using values of K ranging from 1 to 6. **B.** Bar plot showing average score variances reaching a maximum at K=3, corresponding to the correct number of conformations present in the dataset. **C.** Maps corresponding to conformations of HIV-1 Env in complex with Ab1 (blue), Ab2 (purple), Ab1 + Ab2 (green), and Noisy class (gray) are shown for K=3 and K=4. The unmasked resolutions for maps obtained using K=4 were 8.5 Å, 10.5 Å, 7.3 Å, and 22.0 Å, respectively.

Our approach can be integrated into existing frameworks for unsupervised single-particle cryo-EM refinement and allow end-to-end, fully unsupervised data analysis from raw data to 3D classification. For validation, we integrated our new unsupervised classification approach with our previously reported automatic particle sorting routines that fully automate the 3D refinement pipeline [44]. We first applied the combined procedure to a “blind” mixture of images obtained by combining micrographs from the three different antibody-bound HIV-1 Env datasets used above, Figure 3A. In this case, raw micrographs from the three datasets were combined into one large dataset containing a total of 2,898 micro-graphs. 426,385 particles were automatically detected using a template-free picking method and were extracted using a box size of 192 pixels and a binning factor of 2 [45]. Extracted particles were subjected to the 3D refinement pipeline implemented in cisTEM using an initial reference that was low-pass filtered to 40 Å to prevent reference bias. After 4 iterations of global search and 4 iterations of local refinement, 112,531 clean particles were automatically selected based on the bimodal distribution of cisTEM scores [44]. We then applied our unsupervised classification strategy and confirmed that the mean score variance peaked at K=3, coinciding with the correct number of classes present in the mixture, Figure 3B-C. In this case, we also observed that for K=4, we obtained the three expected classes (with slightly better resolution) plus an additional “noisy” class containing low-quality particles that survived the initial score-based cleaning procedure. Since the consensus refinement was done using a mixture of three datasets, it is remarkable that the cleaning procedure based on the bimodal distribution of cisTEM scores was still able to discriminate good particles from bad, and allow the subsequent unsupervised classification analysis. This experiment represents the first demonstration of a fully automated pipeline for 3D classification, starting from raw electron micrographs to high-resolution reconstructions of the correct number of conformations.

**Fig. 4.**
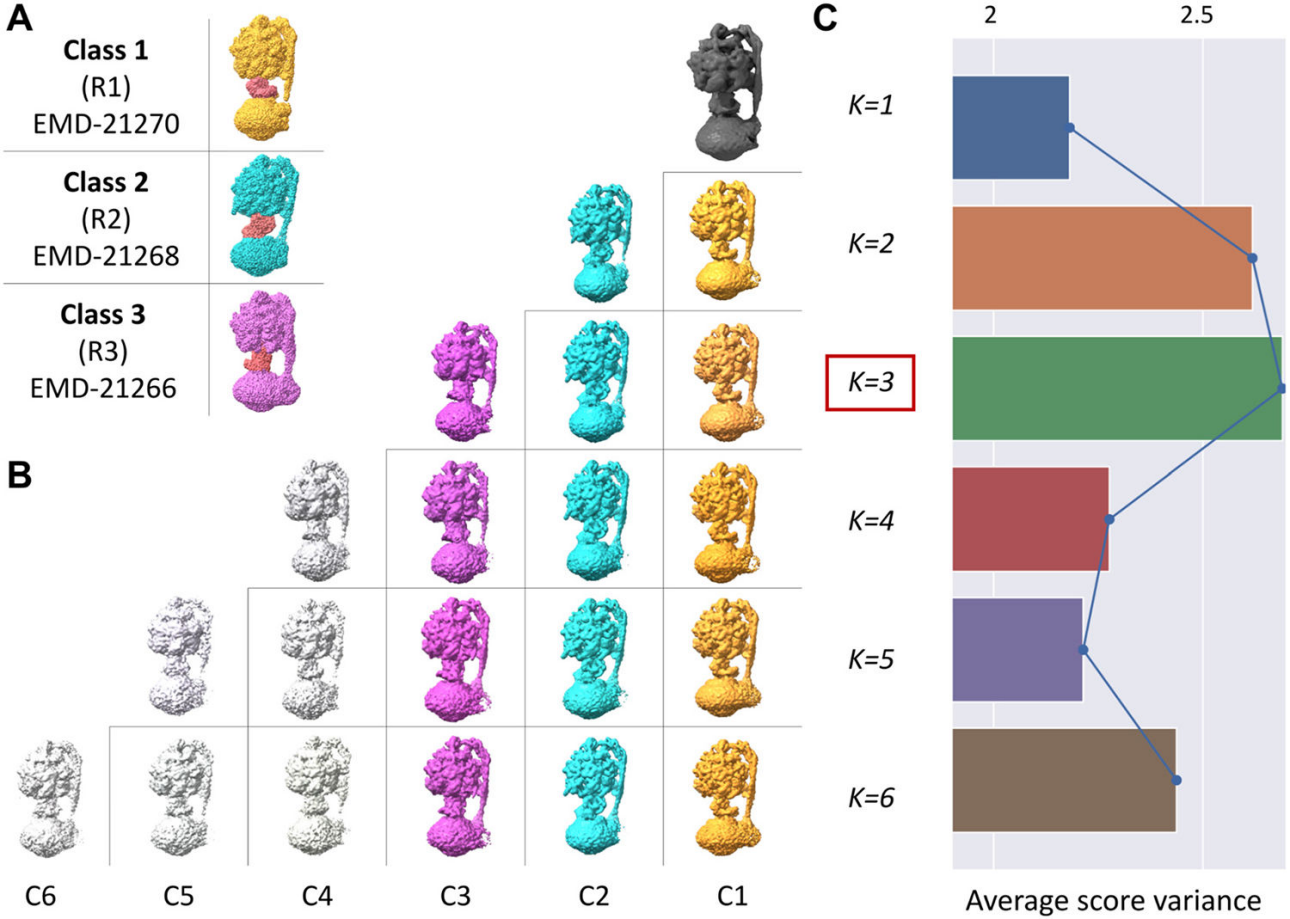
Automatic determination of number of conformations in ATP synthase dataset. **A.** Three low-pass filtered original conformations of ATP synthase obtained from EMPIAR-10475. **B.** Results of 3D classification for K=1−6 are shown as a triangular matrix with each map colored according to the corresponding conformation: Class 1 (golden), Class 2 (cyan), Class 3 (magenta), and Noisy classes (gray). **C.** Distribution of average cisTEM score variances for each value of K. Red box highlights the location of the highest bar, indicating that our approach correctly predicted the presence of three conformations having unmasked resolutions of 6.8 Å (Class 1), 7.5 Å (Class 2), and 7.5 Å (Class 3). While this result confirms the findings of the original study [46], the smaller differences observed between the bars, may indicate the presence of minority intermediate states characteristic of the 360-degree rotational motion of the ATPase synthase rotor.

We also applied our fully automated 3D refinement and classification procedure to micrographs from the Chloroplast ATP synthase complex consisting of 2,063 movies available from EMPIAR-10475 [46]. The three classes present in this dataset correspond to different rotary states of the ATP synthase dynamic complex with the rotor neck being in different orientations relative to the stator. The images were first preprocessed using standard tools for single-particle analysis, including frame alignment with unblur [47] and CTF estimation with CTFFIND4 [48]. Particles were picked using a Gaussian blob and subjected to our iterative refinement and automatic particle sorting procedure [44], using a low-pass filtered version of the original deposited map (EMD-4270) as initial reference. This process resulted in 89,021 clean particles that were automatically selected based on the bimodal distribution of scores. Next, we subjected the clean particles to our unsupervised 3D classification procedure using values of K ranging from K=2−6, Figure 4. The mean score variances in this case, clearly indicated that K=3 was the optimal number of clusters, consistent with the results published in the original study [46]. Differences between the bars in this case are less pronounced than in the HIV-1 Env case, for example, indicating the likely presence of minority intermediate states in addition to the three main ones we detected, which is consistent with the intrinsic structural plasticity of the dynamic ATP synthase complex.

## 4. Discussion

By leveraging the power of computational techniques for 3D classification, single-particle cryo-EM allows the study of heterogeneous complexes at near-atomic resolution. Among the different types of sample variability, this study focuses on the case of discrete heterogeneity where a finite number of conformations is present within the same dataset. Since this type of heterogeneity is very frequently found in everyday applications [5, 49, 50], we expect that our method will be useful and will provide an objective and bias-free strategy for assessing sample heterogeneity. While strategies for 3D classification have been widely used in single-particle analysis, to our knowledge, the problem of automatically determining the number of discrete states present in a dataset has not been successfully addressed. Unlike current subjective strategies that rely on expert-user intervention and are prone to bias, our approach is unsupervised and overcomes the need of routine trial-and-error experiments typically required to determine the optimal partition of a dataset. Our approach represents a simple, yet effective way of automatically determining the number of conformations present in heterogeneous datasets.

Unlike recently proposed classification strategies to determine continuous structural flexibility [13, 14, 36, 37, 51], our method does not assume that particle alignments are known or must remain fixed throughout the clustering analysis. Instead, we determine these parameters during the refinement process and allow every particle to be independently aligned to each reference. This reduces the potential for bias that may be introduced during calculation of the consensus refinement model and does not depend on arbitrary user decisions. It also results in more accurate 3D orientations and translations that in turn translate to higher resolution reconstructions for each conformation. In addition, the observation that noisy classes act as attractors of lower quality particles suggests that using values of K greater than the actual number of conformations present in a mix, may result in further resolution improvements. Since our method is built on top of established tools for 3D classification, it can succesfully handle cases where class-ratios are unbalanced, such as the TRPV5 datasets analyzed above that have a 66/34 mixture ratio and the ATP synthase dataset that has a 54/34/12 mixture ratio. For the same reasons, our method is subjected to the limitations of existing 3D classification routines, including the requirement to have complexes of sufficiently large molecular weight (> 50-75kDa) and a minimum number of particles per class (>10,000 for proteins in the 200-400 kDa range).

An important tool for studying sample heterogeneity is the use of focused refinement techniques that can characterize variability within a confined region specified by a mask [16, 31]. Whether our strategy could be successful in this scenario will depend on the size of the mask and the molecular weight of the focus area, as cisTEM scores are known to become less discriminative when dealing with lower molecular weight complexes [44]. In scenarios where some level of continuous heterogeneity is present, such as the ATP synthase dataset described above, we expect our approach to show score average variances that stay relatively constant for all values of K, indicating that no single discrete partition is better than others. In this case, our clustering strategy will still provide useful information and could lead to the decision to apply tools for studying continuous heterogeneity [13, 14]. Ultimately, our data-driven strategy to automatically determine the number of discrete conformations present in a mix, constitutes an important step towards achieving fully unsupervised and reproducible structure determination workflows in single-particle cryo-EM.

## Supplementary Material

Supplementary Material

## Abbreviations:

TEM: Transmission Electron Microscope
CTF: Contrast Transfer Function
