# Supplementary material for: Data-driven determination of number of discrete conformations in single-particle cryo-EM
Source: Comput Methods Programs Biomed. Author manuscript; Available in PMC 2023 Apr 26. (PMC10131080; doi:10.1016/j.cmpb.2022.106892)
Supplement: Supplementary Material [file NIHMS1890054-supplement-Supplementary_Material.docx]

**SUPPLEMENTARY INFORMATION**

## In-vitro mixing of particles from different datasets

For the HIV-1 Env samples, each dataset was first subjected to the standard single-particle cryo-EM pipeline consisting of movie frame alignment, CTF estimation and particle picking. This was followed by multiple rounds of consensus refinement using our previously reported particle sorting method that automatically identifies the subsets of clean particle images [[44]](https://www.zotero.org/google-docs/?aMrixx). 50,000 particles were extracted from the clean particle stack of each dataset using the newstack command from the IMOD package [[52]](https://www.zotero.org/google-docs/?7YS3Gl), and merged into a single particle stack. The corresponding lines in the .par file containing metadata with alignment and CTF information were also merged and used during processing of the combined stacks. For the TRPV5 datasets, clean particles were downloaded from EMPIAR-10253 (100,540 particles) and EMPIAR-10256 (66,071 particles) and merged into a single particle stack using a similar strategy as above.

## Consensus refinement of combined particle sets

The combined particle stacks were refined against an external map (obtained from one of the datasets) using 3D auto-refinement in cisTEM [[16]](https://www.zotero.org/google-docs/?k6S985). To avoid reference-bias, frequency information corresponding to resolutions higher than 6 Å were excluded for the purpose of image alignment and 8 iterations were performed during the consensus refinement stage.

## 3D classification and statistical analysis

In order to find the correct number of conformations, 3D classification jobs were executed using increasing values of $K$. For a given dataset, the maximum value of $K$ can be estimated based on the total particle number and the sample size [[53]](https://www.zotero.org/google-docs/?e3hliQ). In practice, at least several thousands particles are required to obtain high-resolution structures, depending on particle orientation distribution and symmetry. While values of $K$ higher than 10 are rarely considered in practice [[14]](https://www.zotero.org/google-docs/?vNXyan), the advent of larger datasets and high-throughput data acquisition may soon require using larger $K$.

For 3D classification in cisTEM, each run was initialized using the same consensus refinement parameters. Frequency information corresponding to resolutions higher than 8 Å was not used for the purpose of image classification to prevent overfitting. During classification, Euler angles were refined locally every 3 iterations, and particle positions every 2 iterations. Only a circular mask that included the entire particle density was applied. 22 iterations were needed for the classification runs to converge and this number was kept constant for all datasets. In all cases, we confirmed that noisy classes always contained particles from all component datasets, indicating that differences in imaging conditions do not affect the outcome of the classification. Data processing statistics for all datasets analyzed in this study are shown in **Supplementary Table 1**.

##

## Automatic processing of chloroplast ATP synthase dataset (EMPIAR-10475)

2,063 motion corrected averages were downloaded from EMPIAR-10475 and used as input to our algorithm. CTF estimation was performed using CTFFIND4 [[48]](https://www.zotero.org/google-docs/?I85tHa). 538,354 particles were automatically picked using a template-free method [[45]](https://www.zotero.org/google-docs/?ilz0lU), extracted using 96x96 pixel boxes (binning factor of 4) and subjected to our unsupervised sorting procedure using a reference from EMD-4270 low-pass filtered to 30 Å. 89,021 clean particles were automatically selected according to the bimodal score-distribution criteria [[44]](https://www.zotero.org/google-docs/?aSMGG9). Clean particles were then re-extracted using a box size of 192 pixels (binning factor of 2), and subjected to refinement in cisTEM using 8 additional iterations of local parameter search (doing global searches did not result in measurable improvements in alignment accuracy and were much slower to execute). These refinement results were used to initialize multiple 3D classification runs using values of $K$ ranging from 2 to 6. In each case, 22 refinement iterations were performed using the 3D classification job implemented in cisTEM. Scores corresponding to the last iteration of each run were recorded and used to calculate the mean score variance plots in order to derive the optimal number of clusters present in each mix.

**
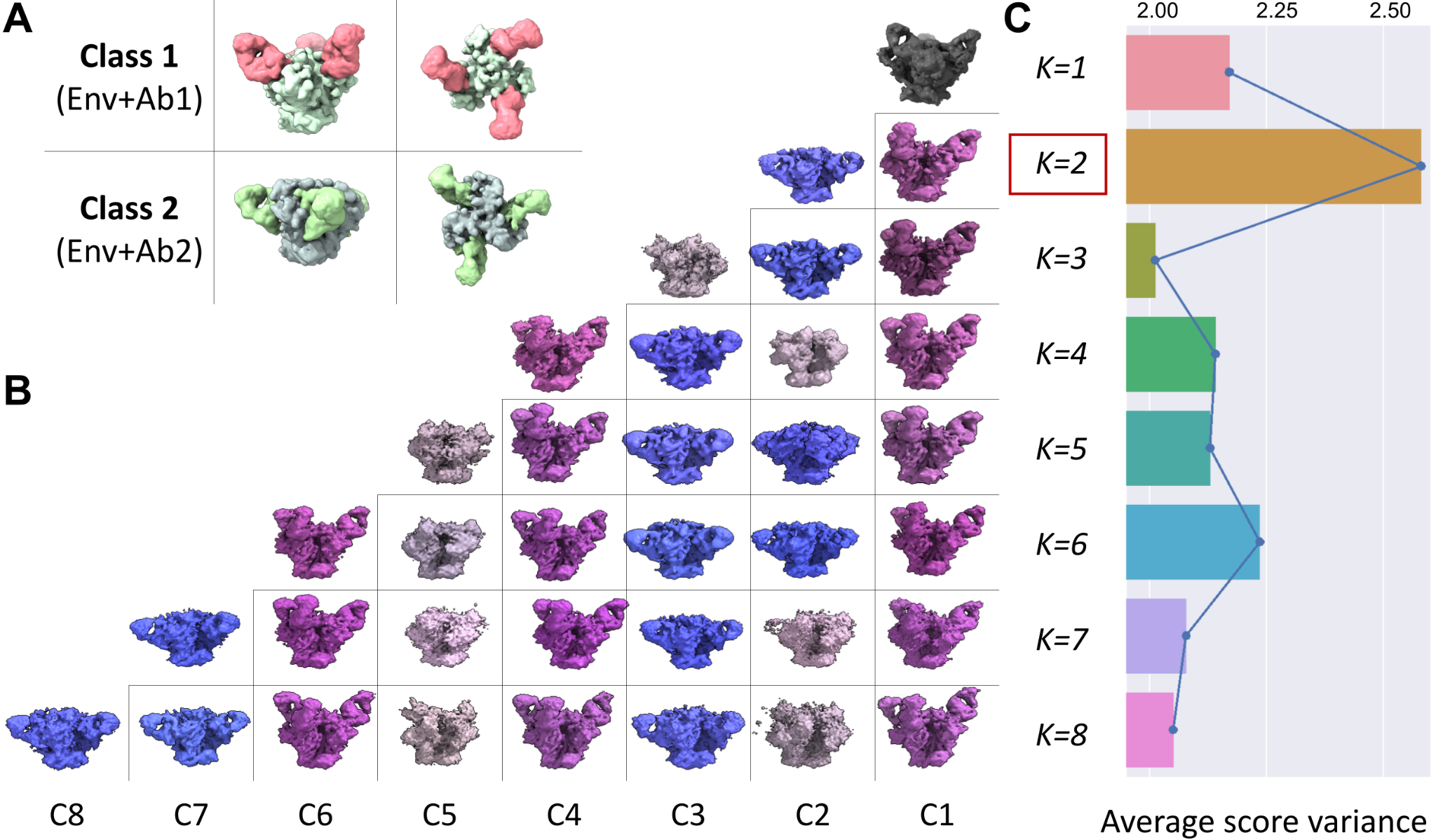
**

**Supplementary Figure 1. Automatic determination of the number of HIV-1 Env conformations from a mixture of two Ab-bound species.** **A**. Two original conformations corresponding to the Env bound to Ab1 (Class 1) and Ab2 (Class 2). Density for Ab1 is shown in light red and density for Ab2 is shown in light green. **B**. Incremental classification results are shown as a triangular table where each row represents 3D classification results obtained for values of $K$ ranging between 1 (consensus model) and 8. Reconstructions are color coded according to their identity: Consensus map (gray), Class 1 (purple), Class 2 (blue), and Noisy classes (pink), which appear only for $K=3-8$. **C**. Bar plot on the right shows the distribution of cisTEM average scores variances for each value of $K$. Red box highlights the peak occurring at position $K=2$, coinciding with the number of different species present in the mix.

**
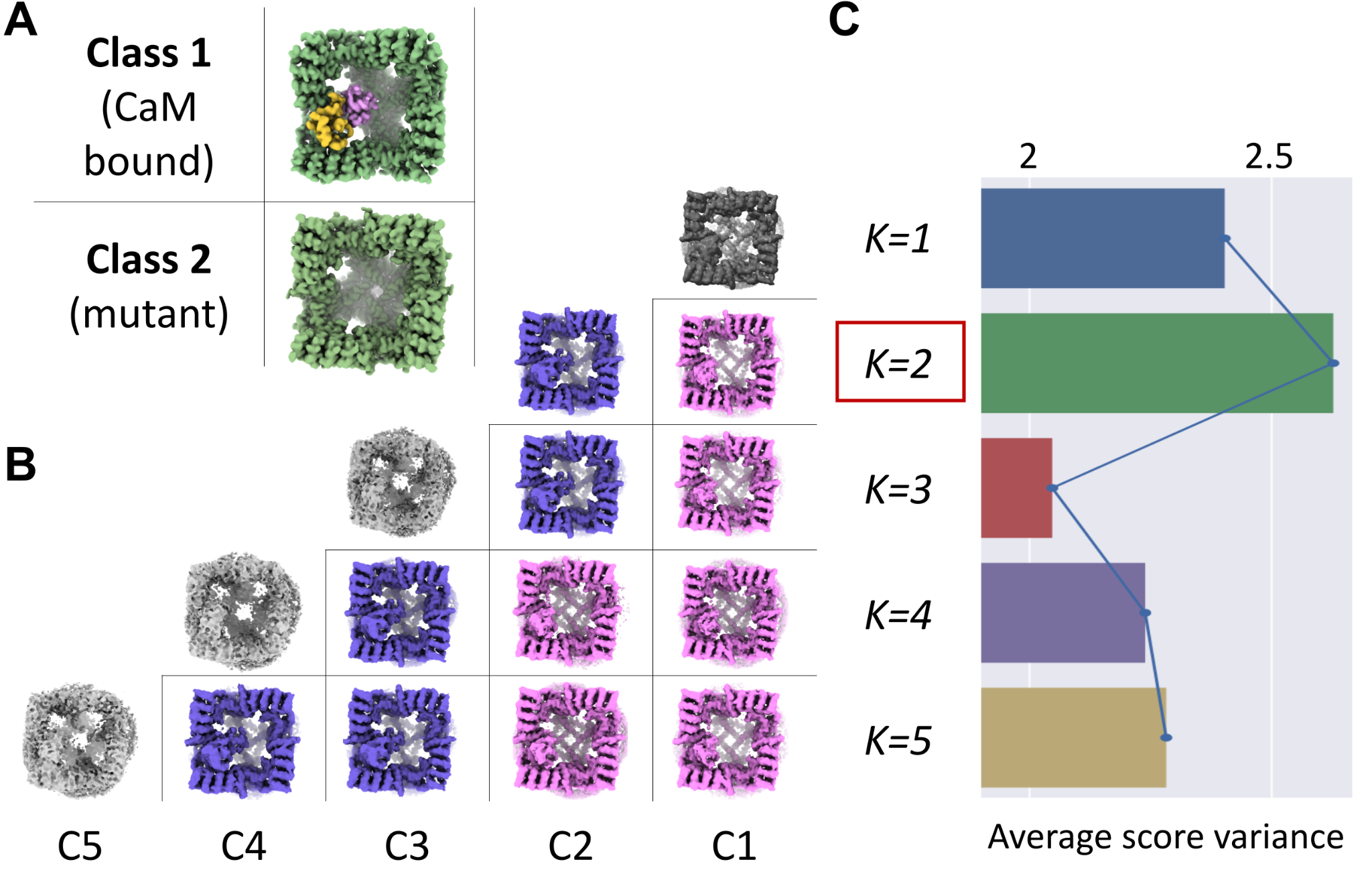
**

**Supplementary Figure 2. Unsupervised classification of TRPV5 dataset (EMPIAR-10253, EMPIAR-10256)**. **A**. Low-pass filtered original reconstructions of the CaM-bound channel and the TRPV5 mutant class. **B**. Triangular table shows the reconstructions obtained during 3D classification from $K=1$ (consensus) to $K=5$. Maps are colored according to class identity: Class 1 (purple), Class 2 (pink), and Noisy class (gray). **C**. Bar plot of average variance cisTEM scores is shown on the right for each value of $K$. The maximum in this case is also achieved when $K=2$ (red box), which coincides with the actual number of conformations present in the mix.

**
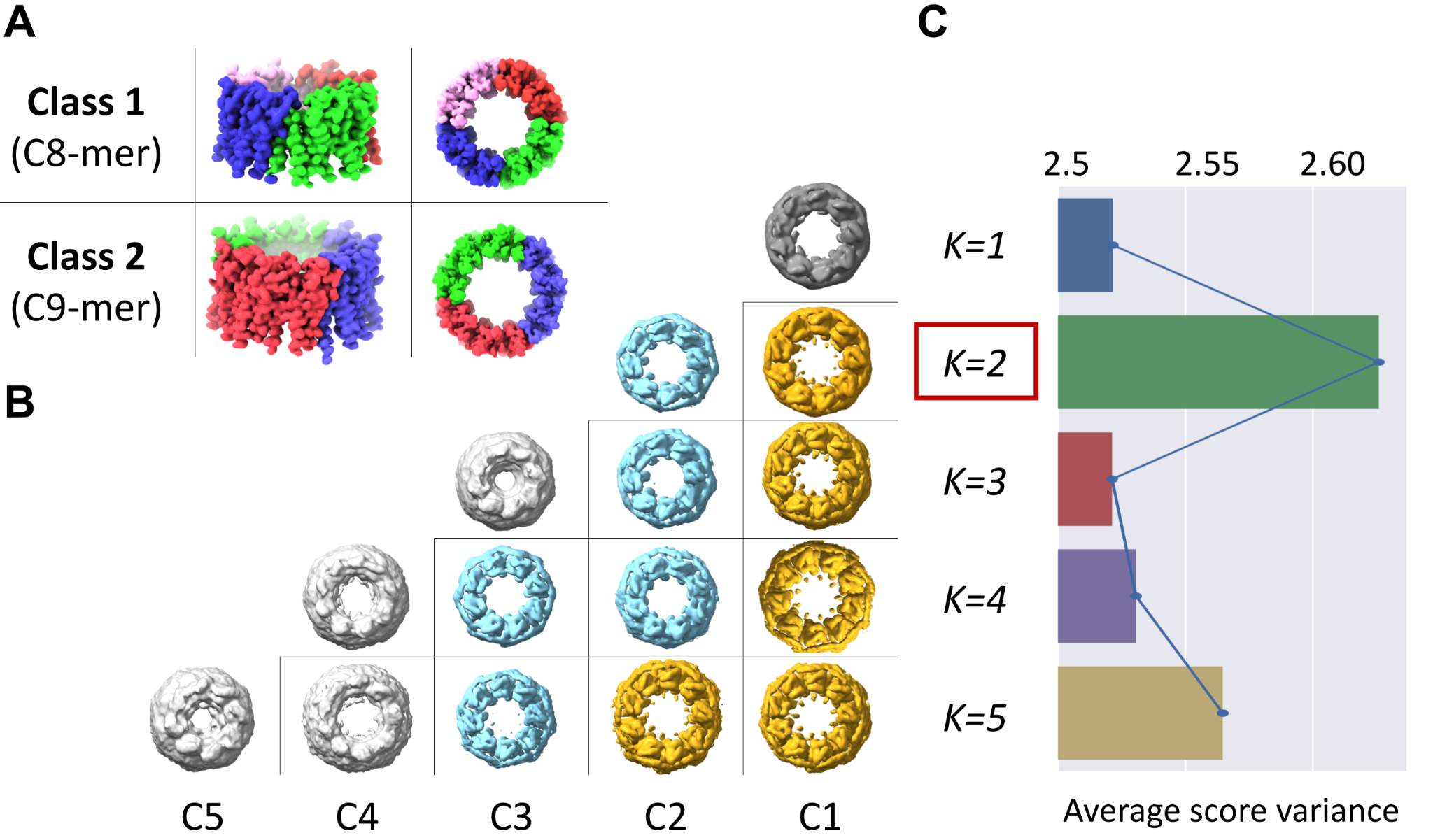
**

**Supplementary Figure 3. Automatic classification of calcium homeostasis modulator (CALHM) channels**. **A**. Two low-pass filtered original reconstructions for the C8 symmetry 8-mer (Class 1) and the C9 symmetry 9-mer (Class 2). **B**. Triangular matrix shows incremental classification results for particles extracted from EMPIAR-10444 using $K=1$ (consensus model) to $K=5$. Reconstructions are colored according to the class identity: Class 1 (gold), Class 2 (cyan), and Noisy class (gray). **C**. Bar plot on the right shows average cisTEM variance scores for each value of $K$, with the maximum reached when $K=2$, coinciding with the correct number of conformations present in the dataset.

| **Sample name** | **Conformations**  **present** | **Microscope/Camera/**  **Pixel size (Å)** | **Number of micrographs** | **Number of particles**  **(occupancy)** | **Resolu-**  **tion (Å)** |
| --- | --- | --- | --- | --- | --- |
| HIV-1 Env trimer | Env + Ab1  Class 1 | Krios/K3/  1.066 | 1066 | 50,000 | 4.3 |
|  | Env + Ab2  Class 2 | Krios/K3/  1.066 | 934 | 50,000 | 4.0 |
|  | Env + Ab1/2  Class 3 | Krios/K3/  1.066 | 898 | 50,000 | 3.0 |
| TRPV5 channel | EMPIAR-10256  Class 1 | Krios/K2/  1.059 | N/A | 66,071 (34%) | 3.3 |
|  | EMPIAR-10253  Class 2 | Krios/K2/  1.059 | N/A | 100,540 (66%) | 2.8 |
| ATP synthase | EMPIAR-10475 | Krios/K2/  1.053 | 2063 | 86,111 (100%) |  |
|  | Class 1  Class 2  Class 3 |  |  | 46,180 (54%)  29,667 (34%)  10,264 (12%) | 3.3  4.0  4.3 |
| CALHM channel | EMPIAR-10444 | Krios/Falcon III/0.83 | 2808 | 461,347 (100%) |  |
|  | Class 1  Class 2 |  |  | 233,566 (51%)  227,781 (49%) | 3.4  3.4 |

**Supplementary Table 1.** **Data processing statistics of cryo-EM images analyzed in this study**. For each dataset, we report data acquisition parameters, number of micrographs, number of clean particles assigned to each conformation, and corresponding final refinement resolutions according to the 0.143-FSC between half-maps.

# 
